# Analysis of a Cell Wall Mutant Highlights Rho-Dependent Genome Amplification Events in Staphylococcus aureus

**DOI:** 10.1128/spectrum.02483-21

**Published:** 2022-09-12

**Authors:** Raquel Portela, Nuno A. Faria, Michael Mwangi, Maria Miragaia, Hermínia de Lencastre, Alexander Tomasz, Rita Gonçalves Sobral

**Affiliations:** a Associate Laboratory i4HB - Institute for Health and Bioeconomy, NOVA School of Science and Technology, Universidade NOVA de Lisboa, Caparica, Portugal; b Laboratory of Molecular Microbiology of Bacterial Pathogens, UCIBIO – Applied Molecular Biosciences Unit, Department of Life Sciences, NOVA School of Science and Technology, Universidade NOVA de Lisboa, Caparica, Portugal; c Laboratory of Bacterial Evolution and Molecular Epidemiology, Instituto de Tecnologia Química e Biológica António Xavier, Universidade Nova de Lisboa (ITQB NOVA), Oeiras, Portugal; d Laboratory of Microbiology and Infectious Diseases, The Rockefeller University, New York, New York, USA; e Laboratory of Molecular Genetics, Instituto de Tecnologia Química e Biológica António Xavier, Universidade Nova de Lisboa (ITQB NOVA), Oeiras, Portugal; Emory University School of Medicine

**Keywords:** antimicrobial resistance, cell wall, DNA recombination, methicillin resistant *Staphylococcus aureus*, Rho termination of transcription factor

## Abstract

In a study of antibiotic resistance in Staphylococcus aureus, specific cell wall mutants were previously generated for the peptidoglycan biosynthesis gene *murF*, by the insertion of an integrative plasmid. A collection of 30 independent mutants was obtained, and all harbored a variable number of copies of the inserted plasmid, arranged in tandem in the chromosome. Of the 30 mutants, only 3, F9, F20 and F26, with a lower number of plasmid copies, showed an altered peptidoglycan structure, lower resistance to β-lactams and a different loss-of-function mutation in *rho* gene, that encodes a transcription termination factor. The *rho* mutations were found to correlate with the level of oxacillin resistance, since genetic complementation with *rho* gene reestablished the resistance and cell wall parental profile in F9, F20 and F26 strains. Furthermore, complementation with *rho* resulted in the amplification of the number of plasmid tandem repeats, suggesting that Rho enabled events of recombination that favored a rearrangement in the chromosome in the region of the impaired *murF* gene. Although the full mechanism of reversion of the cell wall damage was not fully elucidated, we showed that Rho is involved in the recombination process that mediates the tandem amplification of exogeneous DNA fragments inserted into the chromosome.

**IMPORTANCE** The cell wall of bacteria, namely, peptidoglycan, is the target of several antibiotic classes such as β-lactams. Staphylococcus aureus is well known for its capacity to adapt to antibiotic stress and develop resistance strategies, namely, to β-lactams. In this context, the construction of cell wall mutants provides useful models to study the development of such resistance mechanisms. Here, we characterized a collection of independent mutants, impaired in the same peptidoglycan biosynthetic step, obtained through the insertion of a plasmid in the coding region of *murF* gene. S. aureus demonstrated the capacity to overcome the cell wall damage by amplifying the copy number of the inserted plasmid, through an undescribed mechanism that involves the Rho transcription termination factor.

## INTRODUCTION

Since early studies, knowledge on the mechanisms of beta-lactam resistance in Staphylococcus aureus has evolved concomitantly with the study of cell wall biosynthesis.

Previous work on *murF* gene included the construction and characterization of an insertion and a conditional mutant, both constructed in the background of methicillin-resistant Staphylococcus aureus (MRSA) strain COL (1, 2). The conditional mutant COLspac*murF*, in which *murF* was under the control of an inducible promoter, demonstrated that *murF* is essential for S. aureus. For suboptimal levels of *murF* expression, the tripeptide accumulated in the peptidoglycan in an inversely proportional way, while the growth rate and the oxacillin resistance level, decreased in direct accordance (2).

The *murF* insertion mutant F9 resulted from the insertion of pRS2, a suicide vector, constructed by cloning of 1057 bp of the terminal region of *murF* internal fragment until the 6 nucleotides before the *murF* stop codon, into pSP64E plasmid, and showed the same altered peptidoglycan profile and decreased resistance to oxacillin.

The pRS2 plasmid inserted into F9 chromosome in the form of 6 tandem copies, as previously determined by restriction mapping (1). The percentage of disaccharide tripeptide accumulated in the peptidoglycan of F9 was approximately 9% but the strain was able to grow and divide properly, showing no decrease in fitness – in contrast to the conditional mutant.

In this work, whole genome sequencing of mutant F9 allowed to identify a single mutation in *rho* gene and to establish a link between the integrity of this transcription termination factor, the number of pRS2 copies inserted in the chromosome of the *murF* mutants and the level of resistance to beta-lactams.

Rho is a RNA translocase/helicase from the RecA-family, responsible for transcription termination at specific chromosomal locations (3) by a three-step mechanism: Rho binds to the mRNA molecule, translocates across it until it reaches the RNA polymerase (RNAP) and the transcript is released (4).

Although scarce information exists on S. aureus Rho protein, besides not being essential (5), several roles have been assigned in other bacteria. In Escherichia coli, Rho mediates the release of obstructing transcription-elongation complexes to allow the passage of the replisome, thus maintaining chromosome integrity (6). Additionally, Rho prevents the accumulation of R-loops - three-stranded hybrid structures of DNA:RNA that form as the nascent transcript binds to the transiently exposed DNA template upstream of the elongation complexes. Formation of R-loops promotes strand breaks in the DNA and consequently, genome instability, which may prevent the replication fork to proceed (7–9).

Another major role of Rho is to suppress pervasive transcription that frequently originates non-coding transcripts not demarked by gene boundaries and mainly antisense. Pervasive transcription seems to arise from spurious promoters and the generated transcripts may have regulatory functions and therefore must be tightly regulated (10).

Rho is also involved in transcription-translation coupling, preventing transcriptional polarity. When the level of translation of a mRNA molecule is low, Rho stops its transcription, affecting the expression of the downstream genes in the same operon (11).

Rho is also associated with the protection of cells from exogenous DNA in E. coli. Several prophages and horizontally transferred genes have a Rho dependent termination mechanism that tightly regulates the transcription of these acquired genes, that can offer benefits, but can also present a menace (12). However, this role may not be universal since in Bacillus subtilis, lack of Rho does not induce the expression of prophage-related genes (13).

Besides the functions already attributed to Rho, we describe yet another role in the amplification, by recombination, of a plasmid inserted in the chromosome. We demonstrate that Rho is responsible for the capacity of the *murF* insertion mutants to overcome a cell wall damage that compromises the fitness and resistance to beta-lactams of S. aureus.

## RESULTS

**FIG 1:**
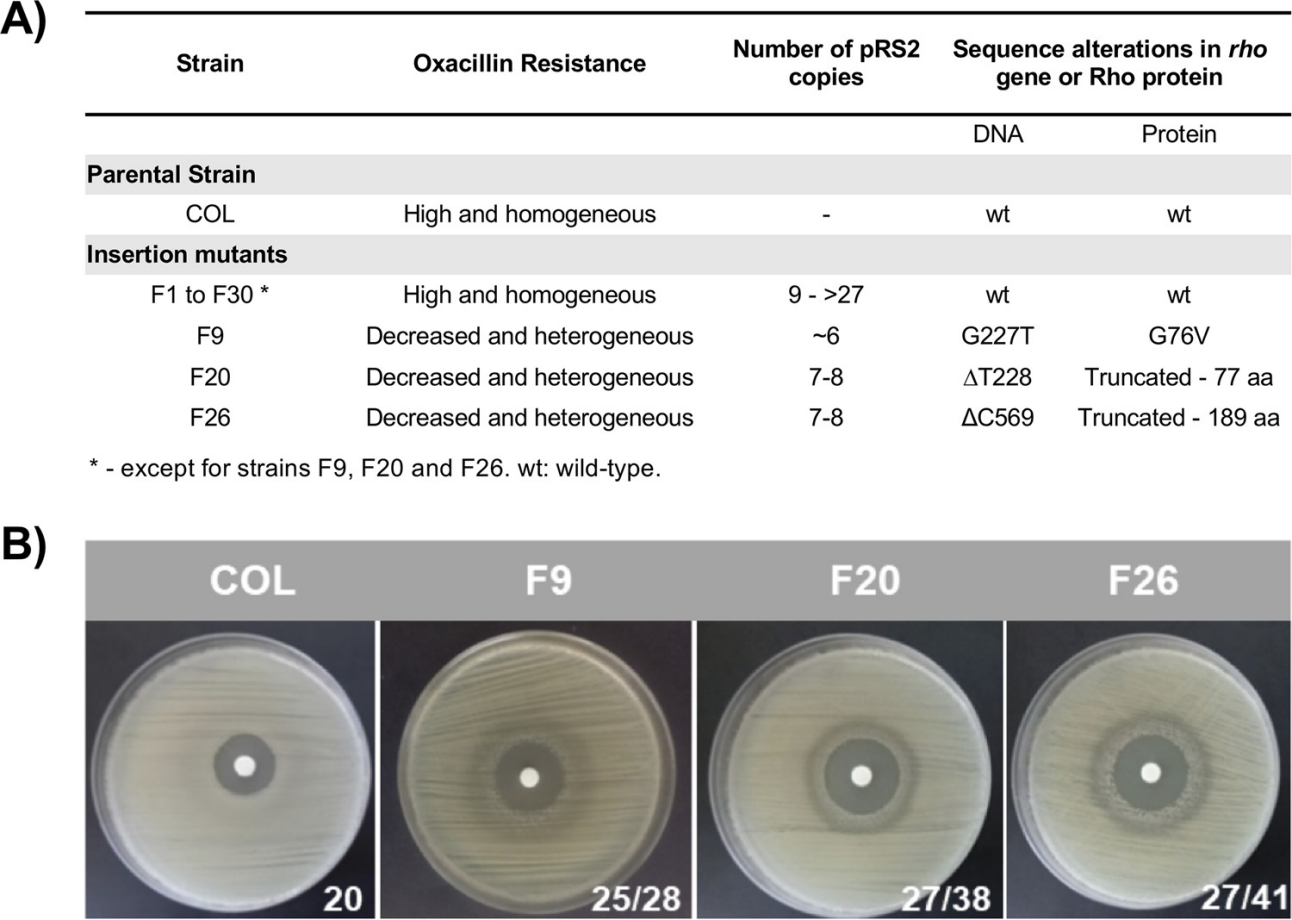
Phenotypic and genotypic characteristics of the insertion mutants. (A) Oxacillin resistance phenotype, number of pRS2 copies in the chromosome and presence of alterations in the sequence of *rho* gene and Rho protein. (B) Growth inhibition halos for oxacillin (1 mg) obtained by disk diffusion for the parental strain COL and the susceptible insertion mutants F9, F20 and F26. The values presented correspond to the halo diameter in mm. The 2 values of diameter of the halos correspond to the presence of resistant subpopulations.

A *murF* insertion mutant was obtained by electroporation of pRS2 integrative plasmid into RN4220 S. aureus strain, that integrated into the chromosome by homologous recombination through the cloned 1057 bp *murF* fragment (1). The plasmid integration region was then transduced into the MRSA strain COL and 30 independent transductants were isolated. Among these, only 3 mutants, F9 (1), F20 and F26, showed a decreased level of oxacillin resistance. The remaining mutants showed high level resistance to oxacillin, comparable to the parental strain COL (Fig. 1A and B). The peptidoglycan of strain F9 showed accumulation of tripeptide monomer (1), while mutant F1, as a representative of the high level oxacillin resistance group, showed parental-like peptidoglycan (Fig. S1).

### Transcription level of *murF* of the insertional mutants.

**FIG 2:**
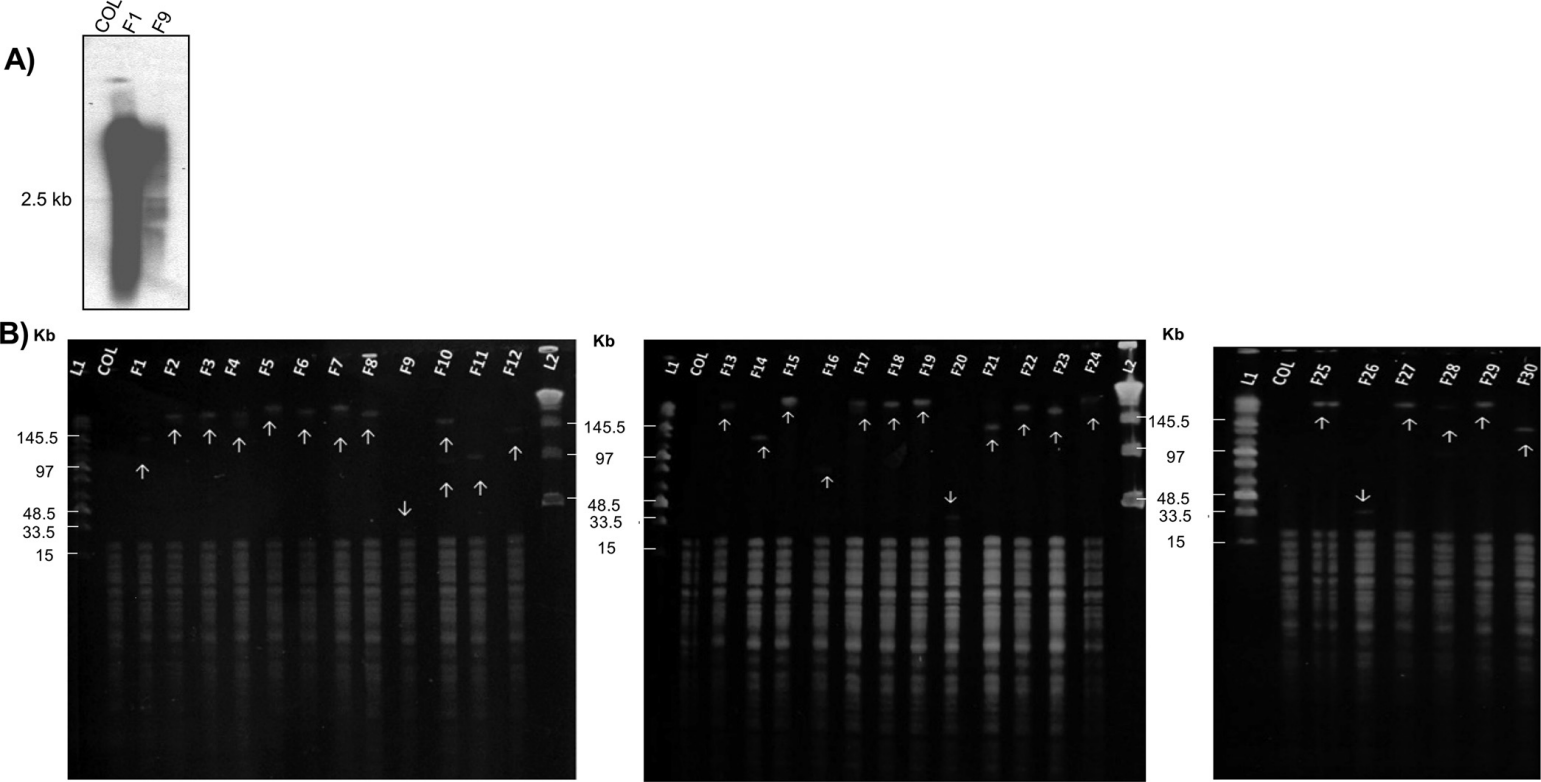
*murF* transcription analysis and pRS2 copy number determination in S. aureus *murF* insertional mutants. (A) Northern blotting of strain COL and the insertion mutants F1 and F9 with a *murF* ^32^P radiolabeled specific probe. (B) Restriction mapping of the 30 pRS2 insertion mutants. Total DNA of COL and the insertion mutants was digested with EcoRV and separated by Pulsed Field Gel Electrophoresis (PFGE). Left panel - strain COL and mutants F1 to F12; middle panel - strain COL and mutants F13 to F24; and right panel - strain COL and mutants F25 to F30. L1 - Mid Range PFGE Marker (New England Biolabs) and L2 - Lambda ladder PFG marker (New England Biolabs). Arrows indicate the position of the DNA band corresponding to the multiple copies of pRS2 plasmid.

The level of *murF* transcription of strains F1 and F9 (as representatives of a resistant and a susceptible mutant, respectively) was determined by Northern blotting. The DNA probe included the *murF* fragment that was cloned in pRS2 plasmid. The *murF* transcription pattern of COL consisted of a single band (~2.5 Kb), compatible with the co-transcription of *murF* and *ddlA* genes (2). The *murF* transcription profiles of F1 and F9 showed a strong signal smear compatible with the presence of mRNA transcripts of several sizes, with higher intensity for mutant F1 (Fig. 2A). The high number of copies of the plasmid containing the *murF* terminal region, could explain the multiple transcripts and the strong transcription signal.

The number of pRS2 copies in the chromosome of each mutant was determined by restriction mapping. The DNA was digested with EcoRV, which has no restriction sites in the pRS2 plasmid, resulting in a unique DNA fragment with all the copies of the plasmid (Fig. 2B). The molecular weight of this fragment allowed to calculate the approximate number of pRS2 chromosomal copies, which for 27 of the collection mutants was found to be approximately 16 to >28 (F16:~82 Kb, F25:>145.5 Kb). In contrast, fewer copies of pRS2 were determined for strains F9, F20 and F26 (F9: 6 copies [<33.5 Kb], F20 and F26: 7 copies [~33.5 Kb]) (Fig. 1B).

**FIG 3:**
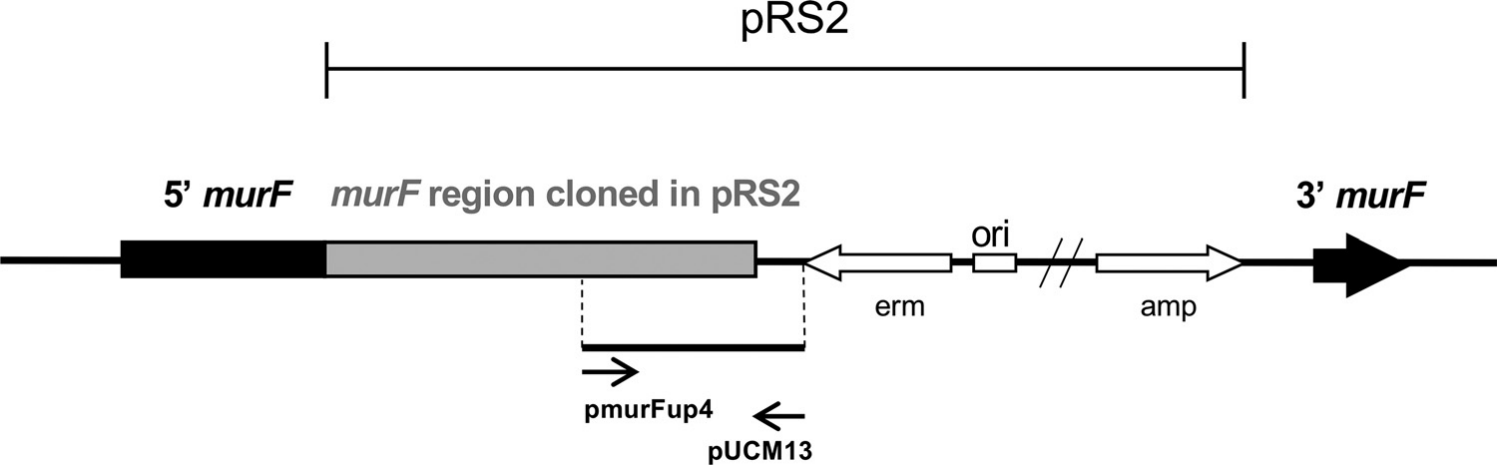
PCR amplification strategy for the truncated end of *murF* gene. Amplification by PCR of the chromosomal region of *murF* gene that was truncated by the insertion of pRS2 plasmid. The *murF* gene is represented by a black truncated arrow, the pRS2 plasmid is represented by white arrows and the internal fragment of the *murF* gene that was cloned in pRS2 plasmid is represented by a gray box. The primers used for the PCR amplification are identified by thin arrows.

To confirm that the 3′region of *murF* gene was correctly disrupted by pRS2, despite the different insertion events that occurred in each of the mutants, this region was analyzed by PCR and sequencing, for strains F1, F10, F16, F20, F26 and F28 (Fig. 3). The last 6 bp of *murF* were replaced by 85 bp of pRS2 plasmid in all strains tested, suggesting that the replacement at 6 bp of the stop codon occurred for all insertion mutants.

Since the *murF* gene is equally impaired in all mutants, the number of plasmids in tandem in the chromosome seems to relate to the different levels of oxacillin resistance observed.

### Whole genome sequencing of insertion mutants F1 and F9.

The genomes of mutants F1 and F9 were compared with the parental strain COL and 6 mutations were identified (Table 1). Two mutations were exclusive of F9, one was a substitution in position 227 of *rho* gene (SACOL_RS11055) that should result in the substitution of glycine 76 for a valine residue (G76V) in Rho protein. The other mutation was a substitution in position 915 of the coding region of DEAD/DEAH box helicase gene (SACOL_RS10840) that resulted in a synonymous substitution (Y305Y). For this reason, this mutation was not further considered. Four other mutations were common to the genomes of F1 and F9 mutants. Two of these were located in the region of *murF* that was repeated in tandem (Table 1). One of the mutations resulted in E125D substitution and the other in E192G substitution.

**TABLE 1:** List of polymorphisms identified in F1 and F9 mutants in comparison to S. aureus parental strain COL by whole-genome sequencing

Mutant	Genome position	Reference	Alteration	Gene	SACOL iD	New locus tag	Protein
F1 and F9	2036233	T	C	hypothetical	1972	RS10310	No alteration
F1 and F9	2140264	T	C	*murF*	2073	RS10845	E125D
F1 and F9	2140464	T	A	*murF*	2073	RS10845	E192G
F9	2137992	G	A	DEAD/DEAH box helicase	2072	RS10840	No alteration
F9	2173340	C	A	*rho*	2113	RS11055	G76V
F1 and F9	2333678	A	T	*modA*	2272	RS11945	C49S

The other 2 mutations common to F1 and F9 were: (i) a mutation in a gene of unknown function, SACOL1972 (SACOL_RS10840), which does not result in a protein alteration, and (ii) a mutation at position 145 of *modA* gene (SACOL_RS11945) which results in C49S protein substitution.

The analysis of the whole-genome sequencing (WGS) results highlighted the mutation in *rho* gene as the only genetically significant difference between the 2 strains, F1 and F9, besides the number of integrated pRS2 copies.

### The *rho* gene is impaired in F9, F20 and F26 *murF* mutants.

The G76 residue of Rho protein that is altered in F9 strain is adjacent to phenylalanine F77. The corresponding residue in E. coli, F64, is directly involved in the interaction with RNA molecules (14) (Fig. S2). The G76V substitution could influence the interaction between Rho protein and RNA molecules, anticipating the impairment of Rho in F9 mutant.

The *rho* gene was sequenced for the other 2 *murF* mutants with decreased resistance to oxacillin (F20 and F26) and for F1 mutant (oxacillin high level representative). Strains F20 and F26 showed 2 different mutations, while the F1 resistant mutant showed no alteration. In mutant F20, a non-sense mutation was found and should result in a premature stop codon and consequently a truncated Rho protein with 77 of the native 438 residues. In mutant F26, a frameshift mutation should result in a truncated Rho protein with 183 of the native 438 residues and 6 extra residues (Fig. 1).

### Complementation with *rho* gene reestablishes the oxacillin resistance phenotype of F9, F20 and F26 mutants.

To analyze the impact of the mutations in *rho* gene, mutants F9, F20 and F26 were complemented with a replicative plasmid carrying the wild-type *rho* gene under the control of a cadmium inducible promoter, generating strains F9+pBCB8*rho*, F20+pBCB8*rho* and F26+pBCB8*rho*.

**FIG 4:**
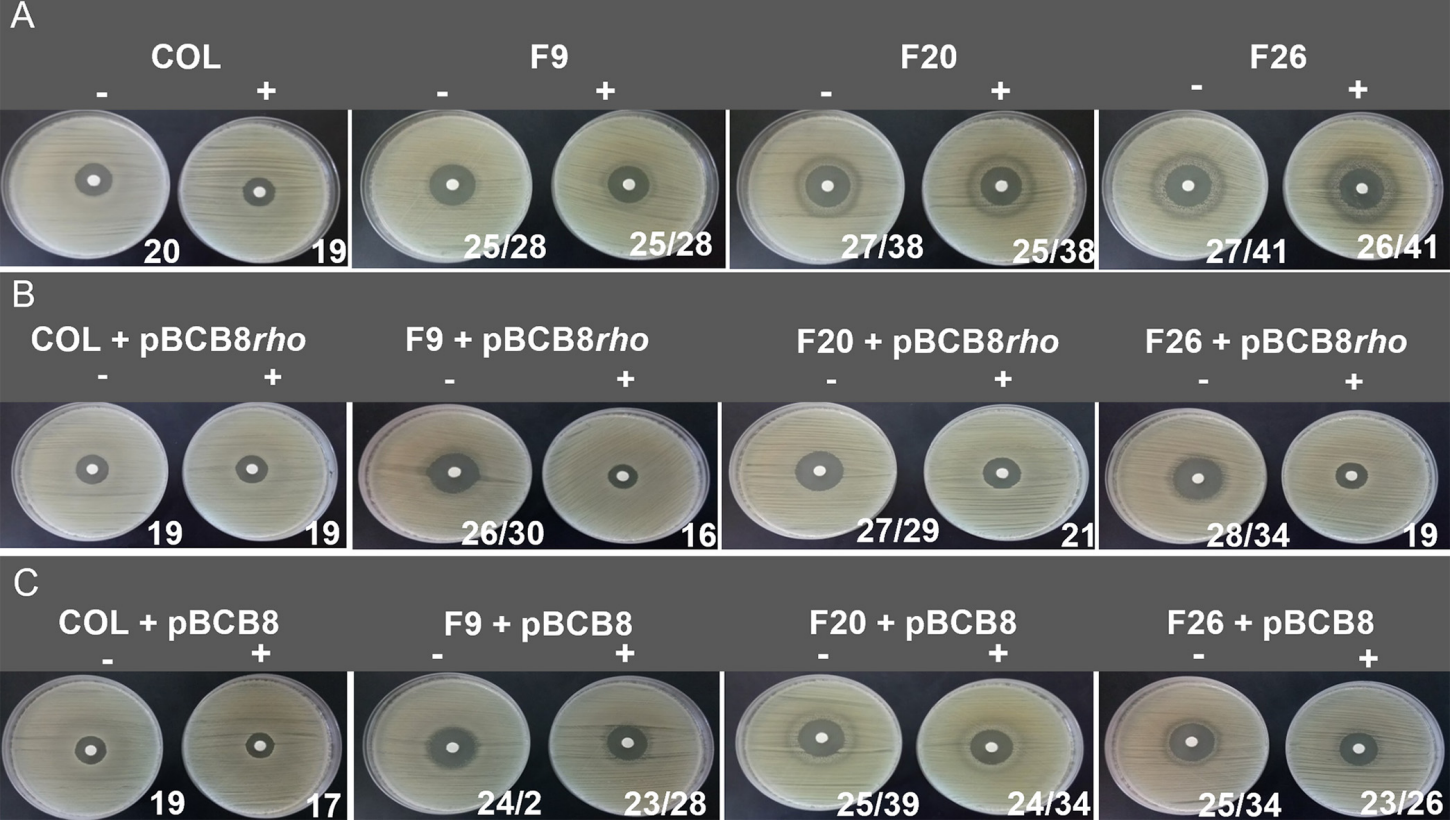
Analysis of oxacillin resistance of *rho* complementation mutants. Growth inhibition halos of oxacillin (1 mg) obtained by disk diffusion. (A) Parental strain COL and the *murF* mutants F9, F20 and F26. (B) Parental strain COL and the *murF* mutants F9, F20 and F26 with the replicative plasmid pBCB8*rho*, carrying the *rho* gene under the control of the inducible pcad promoter. (C) Parental strain COL and the *murF* mutants F9, F20 and F26 with the empty replicative plasmid pBCB8. The values presented correspond to the halo diameter in mm in the absence (-) of the inducer and in the presence (+) of 1 μM CdCl_2_. The 2 values of diameter of the halos correspond to the presence of resistant subpopulations.

In the presence of inducer, the growth inhibition halos of the complemented mutants were restored to the value of the parental strain COL (Fig. 4A and B), while the resistance of parental strains alone and complemented with the empty pBCB8 were not affected (Fig. 4A and C). This successful reestablishment of the high resistance phenotype suggests that impairment of Rho resulted, directly or indirectly, in the decrease of oxacillin resistance of F9, F20 and F26 mutants.

### Consequences of *rho* impairment are specific to the insertion mutants and are not related with the level of *murF* transcription.

To understand if *rho* gene is directly involved in the oxacillin resistance mechanism of COL strain, the Tn *bursa aurealis* transposon mutant NE149 (*rho* inactivated) (15), was used. The *rho* transposon insertion was transduced into strain COL and the oxacillin resistance of the mutant was determined by Etest (Range: 0.016-256 μg/mL). The impairment of *rho* gene did not affect the oxacillin high resistance level of COL (Fig. S3A).

To address if this phenotype was due to the high level of resistance of COL, not covered in the Etest (MIC 800 μg/mL), the transposon was transferred to the low-level resistant MRSA strain HDE288 (SCC*mec* type VI, MIC 0.25 μg/mL) (Fig. S3B). Moreover, the interruption of *rho* gene in this strain allowed to reject the involvement of the *mecA* regulatory elements. To discard the implication of *mecA*, the interruption of *rho* was additionally assessed in the COL-S strain, in which the SCC*mec* cassette was excised (16) (Fig. S3C). The inactivation of *rho* gene was shown not to affect the oxacillin resistance level of HDE288 and COL-S (Fig. S3B and C), suggesting that it is not related with the phenotype of decreased resistance of F9, F20 and F26 strains.

The *rho* transposon insertion was also transferred to the *murF* conditional mutant COLpcad*murF* (17). The impairment of *rho* did not alter the oxacillin resistance level, independently of the expression of *murF*, suggesting that the impact of *rho* impairment on the level of resistance to oxacillin was specific of the insertion mutants and not related with the *murF* transcription level (Fig. S3D).

### Reversion of oxacillin resistance induced by complementation with *rho* is independent of PBP2A.

The complementation of F9, F20 and F26 mutants with *rho* gene restored the parental resistance level. To determine if the reversion mechanism is related with the production of PBP2A, membrane fractions of COL and of the mutants F9 and F9+pBCB8*rho* grown in the presence of 1 μM CdCl_2_ were analyzed by Western blotting with an anti-PBP2A antibody (Fig. S4). As a control, the membrane was re-hybridized with an antibody raised against the amidase domain of the Atl autolysin (Fig. S4).

The amount of PBP2A present in the membrane fraction of the mutants was similar to the parental strain COL. The overexpression of Rho in mutant F9+pBCB8*rho* did not alter the expression of PBP2A, which showed that the mechanism through which Rho restored resistance to oxacillin did not rely on PBP2A protein levels. Also, for F9 mutant, no alterations in the PBP2A production level were found in comparison to COL. To determine if the activity of PBP2A was altered between the parental strain and the insertion mutants, inhibition halos for ceftaroline were determined by disk diffusion (Fig. S5). As a control, the ceftaroline susceptible strain ATCC25923 was used. Ceftaroline is a cephalosporin beta-lactam antibiotic that binds with higher affinity to PBP2A and induces an allosteric change in this protein revealing its active site. Both F1 and F9 mutants showed minor decreased levels of resistance to ceftaroline, in comparison to COL (Fig. S5), revealing that PBP2A activity may be slightly impaired in these mutants. However, the Rho-associated mechanism of resistance to oxacillin does not seem to involve the altered activity of PBP2A, since in F1 the resistance to oxacillin was identical to COL although the resistance to ceftaroline remained similar to F9.

### Complementation with *rho* decreases the accumulation of tripeptide in mutant F9.

**FIG 5:**
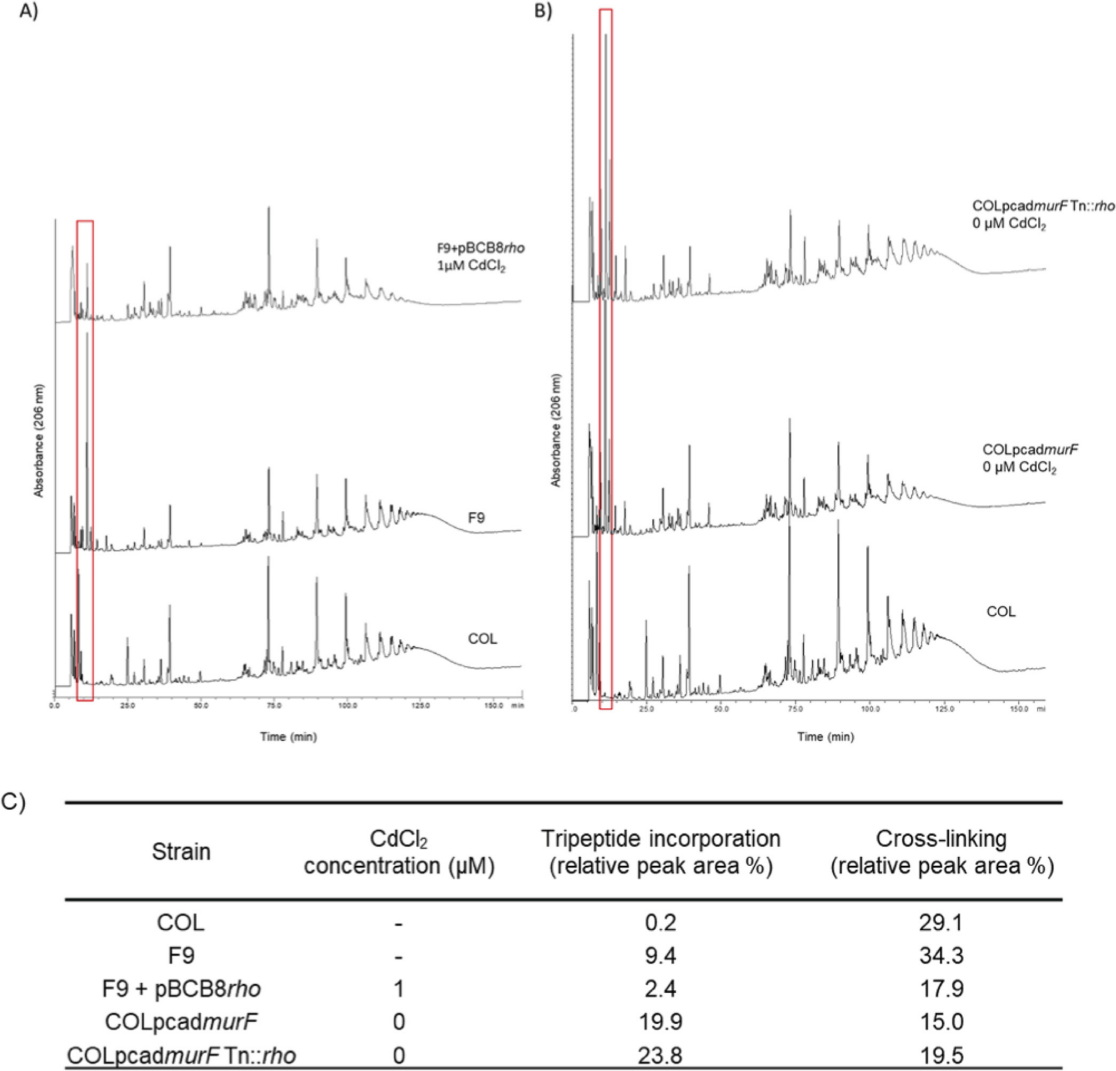
Peptidoglycan analysis of *rho* complementation and transposition mutants. RP-HPLC profiles of purified peptidoglycan digested with mutanolysin (A) Strain COL, F9 *murF* mutant and the complementation mutant F9+pBCB8*rho* grown in the presence of 1 μM of the inducer CdCl_2_. (B) Strain COL, conditional mutant COLpcad*murF* and double mutant COLpcad*murF* Tn::*rho* grown in the absence of inducer. The red box indicates the peaks corresponding to the disaccharide tripeptide (previously identified by mass spectrometry in [2]). (C) Tripeptide incorporation and % of cross-linking of the parental strain COL, the insertion and conditional *murF* mutants and the respective *rho* complementation and *rho* impairment mutants.

To determine if the mechanism of reversion of the insertion mutants includes the repair of the peptidoglycan damage, the peptidoglycan of the parental strain COL, and of mutants F9 and F9+pBCB8*rho* grown in the presence of 1 μM CdCl_2_, was analyzed by RP-HPLC (Fig. 5A). The peaks of the chromatograms were identified by assessing the retention times according to previous works (1, 18) and were integrated. The values are presented as the area percentages of the total area of the chromatogram (Fig. 5C).

Accumulation of tripeptide was observed for mutant F9, as previously (1) with a comparable percentage representation, 9.40% and 8.66%, respectively. For mutant F9+pBCB8*rho* grown in the presence of 1 μM CdCl_2_ (Fig. 5A) the expression of *rho* resulted in the decrease of the accumulation of tripeptide, from 9.40 to 2.40% of relative peak area (Fig. 5C), approximately 75% reduction. This result suggests that the mechanism through which Rho protein reestablishes the oxacillin resistance phenotype of F9 involves the decrease of the abnormal tripeptide accumulation.

### The impairment of *rho per se* does not affect tripeptide accumulation.

To confirm whether the loss-of-function mutation in *rho*, *per se*, affects the level of tripeptide incorporation, we analyzed the peptidoglycan of the double mutant COLpcad*murF* Tn*::rho* that shows tripeptide accumulation, but a different genetic impairment in *murF* (conditional expression) and *rho* gene interrupted by a transposon, mimicking the F9 mutant.

The peptidoglycan of COLpcad*murF*, grown without inducer showed a tripeptide accumulation of 19.9%, comparable to the value of 23.8% observed for the peptidoglycan of COLpcad*murF* Tn*::rho* double mutant grown without inducer (Fig. 5B and C). These results showed that the impairment of *rho* gene does not result in tripeptide incorporation in peptidoglycan.

### Complementation with *rho* increased the number of plasmid copies.

**FIG 6:**
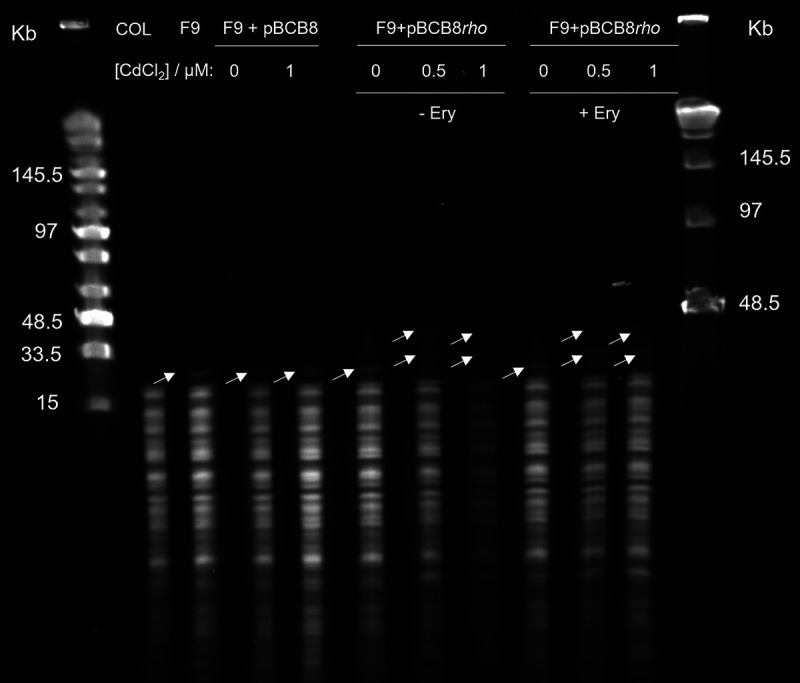
Restriction mapping of *rho* overexpression mutant. Pulsed Field Gel Electrophoresis (PFGE) of total DNA digested with EcoRV, of the parental strain COL, F9, F9+pBCB8 in the absence (0) and in the presence of 1 μM CdCl_2_ and mutant F9+pBCB8*rho* in the absence and in the presence of 0.5 and 1 μM CdCl_2._ F9+pBCB8*rho* was also tested in the absence (- Ery) and in the presence (+ Ery) of erythromycin. Lane 1: Mid Range PFGE Marker (NEB). Lane 12: Lambda ladder PFGE marker (NEB). Arrows indicate the position of the DNA band corresponding to the multiple copies of pRS2 vector.

To address the hypothesis that the mutation in *rho* gene could be involved in the number of copies of pRS2 in the chromosome, restriction mapping with EcoRV, of the complementation mutant F9+pBCB8*rho* grown with and without inducer, was performed (Fig. 6).

The expression of *rho* resulted in the increase of the number of chromosomal pRS2 tandem copies. The band corresponding to the pRS2 copies increased in size, corresponding to an increase from 6 to <10 copies in F9 (Fig. 6).

These results showed that Rho protein is responsible for the tandem amplification of the plasmid in the chromosome. This event was not driven by the presence of the erythromycin resistance gene in pRS2 since the presence of this antibiotic did not influence the number of copies of pRS2 amplified in the chromosome (Fig. 6).

### Insertion mutants present impaired replication.

**FIG 7:**
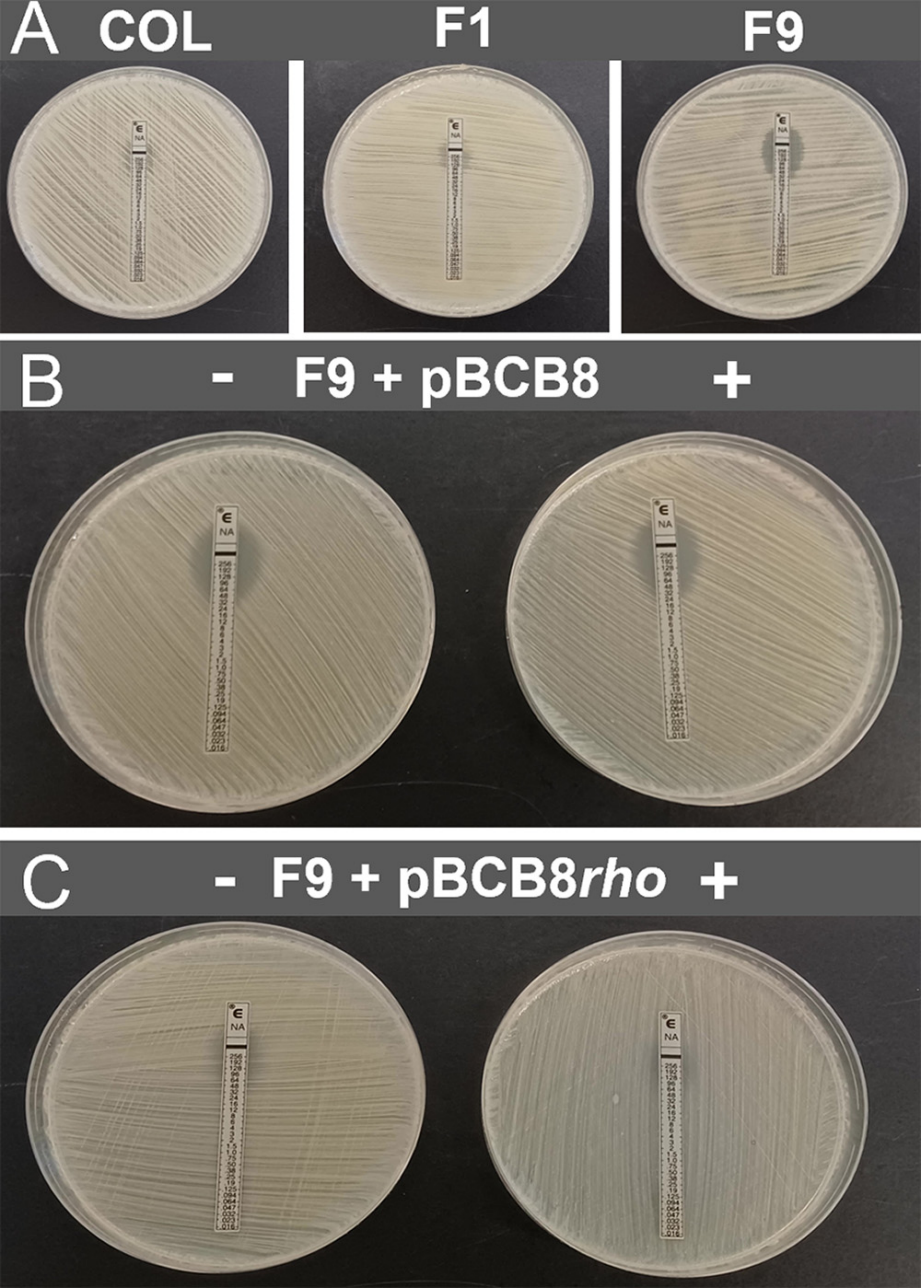
Analysis of nalidixic acid resistance of parental strain, insertion mutants and *rho* overexpression mutants by Etest. (A) strain COL (left panel) and insertion mutants F1 (middle panel) and F9 (right panel); (B) F9 with the empty replicative plasmid pBCB8; (C) F9 with the replicative plasmid pBCB8, carrying the *rho* gene under the control of the inducible pcad promoter pBCB8*rho* in the (-) absence and (+) presence of inducer (0.2 μM CdCl2).

We hypothesized that Rho mediates the tandem amplification of pRS2 during the events of replication of DNA. The high level of transcription that occurs in this genomic region (Fig. 2A), suggests that the balance between transcription and replication of the insertion mutants is impaired and this may be solved by Rho protein. To address if the replication process is affected in these strains, the MIC values for nalidixic acid were determined for the parental strain COL and for the F1 and F9 insertion mutants (Fig. 7A). The strain COL and the mutant F1 presented an MIC of > 256 μg/mL. However, the MIC of mutant F9 was reduced to 32 μg/mL. The decrease in the level of resistance to an antibiotic that targets replication suggests that this process is impaired in mutant F9.

To address the involvement of Rho in the impairment of the replication process in mutant F9, the MIC for nalidixic acid was determined for the complementation mutant F9+pBCB8*rho* in the absence and in the presence of inducer and for the control strain F9+pBCB8 (Fig. 7B and C). Resistance to nalidixic acid was fully reverted to the levels of the parental strain COL, upon *rho* expression. In the absence of the inducer, a slight reversion of the phenotype was already observed for F9+pBCB8*rho*, that may be explained by the leaky nature of the cadmium promoter. The results of mutant F9+pBCB8 corroborated that, in the absence of *rho* gene, resistance to nalidixic acid is not reverted (Fig. 7B). Altogether, these results suggest that the replication process is compromised in the insertion mutants and that Rho is involved in resolving the impairment in this process.

## DISCUSSION

A total of 30 independent mutants were obtained by the insertion of a plasmid in the genome of the MRSA strain COL to disrupt *murF* gene. Two phenotypes were obtained; three mutants showed the expected decrease in the level of resistance to oxacillin (F9, F20 and F26) and the remaining 27 (F1 as representative) showed the same high and homogeneous level of resistance of the parental strain COL. Restriction mapping of the mutants showed that, for all the 30 mutants, the interruption of *murF* gene occurred in the form of several plasmid copies organized in tandem in the chromosome. These plasmid copies were found in higher number in the mutants with high level of resistance to oxacillin and in lower number in the mutants with decreased level of resistance (F9, F20 and F26).

Three different point mutations were identified in the *rho* gene of F9, F20 and F26, and were found to correlate with the level of oxacillin resistance, since only these 3 mutants showed decreased resistance level. Upon genetic complementation with the wild-type *rho* gene, the phenotype of resistance to oxacillin of F9, F20 and F26 was reverted, corroborating the link between Rho and oxacillin resistance in these strains. Moreover, this link was proven to be valid only for the insertion mutants and to be independent of the production and of the activity of PBP2A and to result in a reduction of the abnormal tripeptide in the peptidoglycan. In fact, as demonstrated by the decrease in ceftaroline resistance, the activity of PBP2A is diminished in mutants F1 and F9. However, since Rho is not impaired in F1, and this strain presents the same decrease in ceftaroline resistance, while presents the level of resistance to oxacillin similar to the level of the parental strain COL, we suggest that independently of the mechanism used by Rho to revert the resistance to oxacillin, it is independent of PBP2A expression and activity. Nevertheless, we cannot fully disregard the possibility of delocalization of this protein from the septum in mutant F9, as a consequence of the shortage of the lipid II precursor, promoting the phenotype of decreased resistance to oxacillin.

### Rho is implicated in the tandem amplification of pRS2 plasmid.

The number of pRS2 tandem insertions in the genome of RN4220::pRS2 was >28, corresponding to a DNA fragment of >145.5 Kb. In this strain, that was obtained by plasmid electroporation, the high number of tandem copies of the pRS2 plasmid, should have resulted from a recombination process that occurred after the internalization of several copies of pRS2 or of concatenated plasmids. In RN4220::pRS2, the number of copies of plasmid present were not compatible with the electroporation of multiple copies of the plasmid, due to the low efficiency of this process, nor with the size of the concatemer that was needed to be electroporated.

The pRS2 insertion was then transduced from RN4220::pRS2 into strain COL using phage 80α, that is known to encapsidate DNA fragments of approximately 43.8 Kb (19), corresponding to a maximum of 8 pRS2 copies. Thus, the number of pRS2 tandem copies of the 27 mutants that maintained the phenotypes of COL strain, was not compatible with the transfer of a high number of copies by transduction. This suggests that the transduction event transferred a limited number of plasmid copies, such as those in F9, F20 and F26, corresponding to DNA fragments with a molecular weight lower than 33.5 Kb, compatible with the encapsidation capacity of the phage. Once internalized by COL, the exogeneous DNA fragment integrated into the chromosome, and the pRS2 plasmid was only then amplified in tandem.

In F9, F20 and F26 mutants, for which the tandem amplification of pRS2 plasmid did not occur, *rho* was impaired. Thus, Rho emerged as an essential player in the mechanism of tandem amplification of pRS2, which probably occurred by recombination during chromosome replication.

This hypothesis was confirmed by the analysis of the pRS2 copies in the F9 mutant complemented with *rho* gene. In fact, *rho* complementation triggered the amplification in tandem of pRS2 copies in the chromosome. Although the amplification of resistance determinants upon consecutive antibiotic challenge is a well-documented mechanism of response to a selective pressure (20), the events leading to pRS2 amplification were not triggered by the presence of beta-lactams, nor by erythromycin.

We hypothesize that the amplification of pRS2 copies was mediated by a DNA damage repair mechanism that may rely on the function of Rho as a mediator of transcription-replication conflicts (6). Considering that the replication fork progresses at an approximate rate of 600 nucleotides per second (nts/s) and that the progress rate of the RNA polymerase (RNAP) is around 20 nts/s, transcription-replication conflicts often occur, namely, in highly transcribed regions (6), as is the case of the *murF*::pRS2 region, as demonstrated by Northern blotting of mutants F1 and F9. If these molecular collisions are not properly solved, they could generate fork collapse.

As transcription-elongation complexes are stalled in conflict regions, the backtrack of the RNAP is induced, leading to displacement of the 3′ end of the nascent mRNA and formation of R-loops (21, 22). Additionally, as the RNAP hits the replication fork, double strand breaks or ssDNA gaps are induced and the replication fork cannot progress. Usually, recombination events solve this problem and allow the restart of replication. Rho is described to mediate these conflicts. In E. coli, Rho inhibition induces DNA double strand breaks and formation of R-loops (23). Rho also triggers dissociation of the stalled RNAP, avoiding the transcription-replication conflicts (9) and allowing for the replication to resume or, in case of a DNA lesion, to be repaired. In fact, in Gram-positive bacteria, it has been suggested that Rho was maintained during evolution to prevent conflicts between transcription and replication (6).

We propose that, upon integration of several copies of pRS2 in the chromosome, the high level of transcription of this DNA region has caused transcription-replication conflicts that resulted in DNA breaks, as suggested by the decrease in resistance to nalidixic acid observed for the insertion mutant F9. In the presence of Rho, these conflicts would have been solved by a recombination process that resulted in the amplification of pRS2 copies, which may explain why the resistance to nalidixic acid is not decreased in F1. In mutants in which the *rho* gene was impaired, F9, F20 and F26, this process of recombination did not occur and the number of pRS2 copies reflects the number of copies transduced by the phage.

The variable essentiality and prevalence of *rho* suggests species-specific roles for Rho and alternative pathways of regulation. While in E. coli the abundance of Rho molecules corresponds to 38% to 64% of the abundance of RNAP, in Gram-positive bacteria this ratio is estimated to be 0.8% to 5% (24). Thus, in E. coli Rho may be present at most of the loci where RNAP is catalyzing transcription, while in Gram-positive bacteria, the mechanism of prevention of transcription-replication conflicts must be broader and more indirect such as by inducing a recombination process to repair the damages from the transcription-replication conflicts, that were not properly solved *in loco* due to its reduced abundance.

How the amplification of pRS2 plasmid restored the level of resistance to beta-lactams of the mutants was not elucidated within this work. One possibility is that other PBPs are recruited to compensate for the decreased activity of PBP2A. Additionally, it could be also explained as a direct consequence of the higher number of *murF* truncated copies or by the restoration of one (or more) full *murF* copies during the recombination events. In all the mutants in which there is no recombination (F9, F20 and F26), the *murF* gene is probably translated as a truncated form unable to catalyze the addition of the terminal D-ala-D-ala to the pentapeptide precursor thus accumulating tripeptide at the cell wall and exhibiting decreased resistance to the beta-lactam. This decrease in resistance may be due to the presence of less substrate for PBPs that become more available to bind to oxacillin. The other hypothesis is that the tripeptide promotes the delocalization of PBP2A from the septum. On the opposite side, in the presence of a functional Rho, the events of recombination and amplification of pRS2 may have restored one or more copies of the *murF* gene. In these mutants, a functional MurF would have led to the production and incorporation of the normal pentapeptide at the cell wall, rendering the strain more resistant to the beta-lactam.

However, the role of Rho in resistance could also occur via a more comprehensive mechanism involving the relation between SOS response and resistance. The fitness cost of resistance, namely, in cases in which transcription and translation is affected, is directly linked to the SOS response and to the prevention of R-loops (25). In fact, for all *murF* insertion mutants, the events of transcription and translation are probably uncoupled, and high level resistance is only re-achieved in the presence of a functional *rho* gene, whose product prevents the formation of R-loops.

We plan to identify the role of Rho in DNA recombination by disclosing other players, induced by or acting together with Rho protein. Additionally, we will address the link between the pRS2 tandem amplification and reduction of peptidoglycan damage.

## MATERIALS AND METHODS

### Strains and growth conditions.

Bacterial strains (Table S1) were grown at 37°C with aeration. S. aureus strains were grown in tryptic soy broth or agar (TSB/TSA) (Difco). E. coli was grown in lysogeny broth or agar (LB/LA) (Difco). Erythromycin (10 μg/mL and 5 μg/mL), ampicillin (100 μg/mL), neomycin (50 μg/mL), kanamycin (50 μg/mL), IPTG (100 μM) and cadmium chloride (CdCl_2_) (0.2 and 1 μM) were used as recommended (Sigma).

### DNA methods.

S. aureus DNA was extracted using Dneasy Blood Tissue Kit (Qiagen) as instructed, except for addition of lysostaphin (10 μg/mL) to the lysis buffer.

Routine PCR was performed with NZYTaq polymerase (NZYTech). Amplification for cloning purposes was performed with Phusion High Fidelity polymerase (ThermoFisher Scientific). Digestion and PCR products were purified using NZYGelpure (NZYTech). FastDigest Restriction enzymes were used and ligation reactions were performed using T4 Ligase (ThermoFisher Scientific).

E. coli DH5α was transformed according to (26). Electroporation was performed using Gene Pulser (Bio-Rad) (27) and transduction was performed with 80α phage (28).

### Mutant construction: *murF* insertion mutants.

Insertion mutants F1 to F30 were constructed as described (1) for mutant F9. A 1057-bp *murF* terminal fragment was cloned in pSP64E, using primers pmurFup2 and pmurFdownsalI (Table S2), the later designed at 6 nucleotides upstream from *murF* stop codon.

The resulting pRS2 plasmid was electroporated into strain RN4220 (27) and the correct insertion was confirmed using *murF* primer pmurFup4 and a plasmid primer (pUCM13rev) (Table S2). The construct was transduced from RN4220 into COL (28) and 30 independent transductants (F1 to F30) were isolated.

### Rho overexpression mutants.

The *rho* gene, including the RBS, was amplified from strain COL using primers p93 and p94 (Table S2), with BamHI and EcoRI restriction sites, respectively. The amplification product and the replicative vector pBCB8 were digested and ligated to place *rho* gene under the control of inducible promoter pcad. DH5α competent cells were transformed and plasmid pBCB8*rho* was obtained (Table S1). The construction was sequenced using primers pcadF and p94 (TableS2).

The plasmid was introduced into RN4220 by electroporation and transduced into strains COL, F9, F20 and F26. For control purposes, vector pBCB8 was introduced into the same strains.

### Rho transposition mutants.

The S. aureus NE149 transposition mutant, with Tn *bursa aurealis* inserted in *rho* gene, was obtained from the Nebraska Mutant Library (15). The transposon insertion region was transduced from NE149 into strains COL, COL-S, HDE288 and COLpcad*murF*. The insertion was verified by PCR using primers upstream and buster for the transposon and p50 and p54 for *rho* gene (Table S2), in the 4 possible combinations.

### Restriction mapping.

The number of chromosome-integrated pRS2 copies was assessed by restriction mapping. DNA was extracted in agarose plugs (29), digested with EcoRV, which has no restriction site in pRS2, and separated by Pulsed Field Gel Electrophoresis (initial pulse-1s; final pulse-12s; 6V/cm; 11.3°C; TBE 0.5X). Mid Range PFGE and Lambda ladder PFGE markers were used (New England Biolabs). The approximate number of pRS2 inserted copies was calculated as the ratio between the estimated molecular weight of the band, and the molecular weight of pRS2 (5.2Kb).

### Determination of oxacillin, ceftaroline, and nalidixic acid resistances.

Overnight cultures were washed thrice in fresh medium to remove traces of antibiotic and/or inducer and were swabbed on TSA with the appropriate concentration of the inducer. Oxacillin (Sigma) diffusion disks (1 mg) or ceftaroline (30 μg) were placed on the plates that were incubated at 37°C for 48 h. To determine the oxacillin and nalidixic acid MICs, Etest strips were used (bioMérieux).

### Whole-genome sequencing.

Genomic DNA of COL, F1 and F9 was sequenced with Illumina NextSeq platform. Genomic libraries were prepared using the NexteraXT DNA sample preparation kit (Illumina) and sequenced using 150 bp pair-end reads with 100x estimated coverage. Genomic *de novo* assemblies were performed using INNUca_v3.1 pipeline (30) and polymorphisms detection achieved with CSI Phylogeny_v1.4 (31), using COL as reference (NC_002951.2) and default settings. All the mutations identified in F1 and F9, in comparison with COL, were confirmed through Sanger re-sequencing.

### Northern Blotting.

Cultures were grown to OD_620nm_ 0.7. RNA was extracted as described (2), analyzed by electrophoresis under denaturing conditions (0.66M formaldehyde − 1× morpholinepropanesulfonic acid [MOPS]; Sigma) and blotted onto Hybond N+membranes (Amersham). The *murF* internal probe used was amplified with primers described in Table S2 and labeled with [α-32P]dCTP (Amersham).

### Peptidoglycan purification and analysis.

Peptidoglycan isolation was performed as described (32). Cultures were grown to OD_620nm_ 0.3 and rapidly chilled. Harvested cells were boiled in 4% SDS and disrupted using 106 mm glass beads (Sigma). After treatment with Dnase (10 μg/mL; Sigma), Rnase (50 μg/mL; Sigma), and trypsin (200 μg/mL; Worthington), teichoic acids were removed by incubation with 49% hydrofluoric acid (Merck) for 48 h at 4°C. The peptidoglycan was washed, lyophilized, and digested with mutanolysin (1 mg/mL; Sigma). The muropeptides were reduced with sodium borohydride (Sigma) and separated by RP-HPLC using a Hypersil octyldecyl silane (Runcorn) column in a Shimadzu Prominence system. A linear gradient was used (5% to 30% methanol in 100 mM sodium phosphate, pH 2.5, 0.5 mL/min).

### Purification of membrane fractions.

Cultures were grown to OD_600nm_ 0.7. Cells were washed, resuspended in 50 mM Tris pH 7.5, 150 mM NaCl, 5 mM MgCl_2_ with 0.5 mM phenylmethylsulfonyl fluoride (PMSF) and submitted to freeze-thaw cycles. Lysostaphin (100 μg/mL), lysozyme (50 μg/mL), DNase (10 μg/mL), RNase (10 μg/mL), PMSF (0.5 mM) and β-mercaptoethanol (10 mM) were added and the samples were incubated on ice for 30 min and sonicated. Unbroken cells were removed by centrifugation and the supernatants were centrifuged at 50.000 *g* and washed in 50 mM phosphate buffer, pH 7.0. The membrane fraction was resuspended in 25 mM phosphate buffer pH 7.0, 1% TritonX-100, 10 mM MgCl_2_, 20% glycerol. Total protein was quantified by BCA assay (ThermoScientific).

### Detection of PBP2A.

Membrane preparations (10 μg) were separated by SDS-PAGE (8% acrylamide-0.06% bis-acrylamide) and transferred onto nitrocellulose Hybond-ECL membranes (GE-Healthcare). The membranes were kept on PBS-Tween with 4% low-fat milk overnight and incubated with rabbit anti-PBP2A antibody (raised against peptide NH2-CGSKKFEKGMKKLGVGEDIPSDYPF; RayBiotech) at 1:1000 dilution. After PBS-Tween washes, the membranes were incubated with anti-rabbit HRP-conjugated antibody (PerkinElmer) at 1:5000 dilution. The signal was detected using Western Lightning Plus-ECL (PerkinElmer). The membrane was stripped and re-hybridized with polyclonal antibody against the amidase domain of S. aureus Atl protein, at 1:1000 dilution.

### Data availability.

This Whole Genome Shotgun project has been deposited at DDBJ/ENA/GenBank under the BioProject accession number PRJNA788762, with sample accession numbers JAJQVO000000000, JAJQVP000000000 and JAJQVQ000000000.
