# Supplementary material for: Analysis of a Cell Wall Mutant Highlights Rho-Dependent Genome Amplification Events in Staphylococcus aureus
Source: Microbiol Spectr. 2022 Sep 12;10(5):e02483-21. doi: 10.1128/spectrum.02483-21 (PMC9603463; doi:10.1128/spectrum.02483-21)
Supplement: Supplemental file 1 — Supplemental material. Download spectrum.02483-21-s0001.pdf, PDF file, 0.6 MB [file spectrum.02483-21-s0001.pdf]

Analysis of a cell wall mutant highlights Rho dependent genome amplification events in *Staphylococcus aureus*

Raquel Portela[1,2], Nuno A. Faria[3], Michael Mwangi[4], Maria Miragaia[3], Hermínia de Lencastre[4,5], Alexander Tomasz[4], Rita Gonçalves Sobral[1,2]

SUPPLEMENTAL MATERIAL

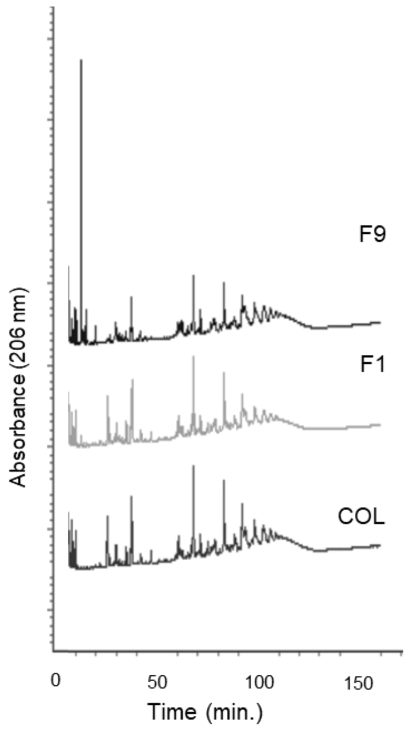

Figure S1. Peptidoglycan analysis of parental strain COL and insertion mutants F1 and F9. RP-HPLC profiles of purified peptidoglycan digested with mutanolysin of parental strain COL and insertion mutant F1 (gray) and insertion mutant F9 (black).

```
CLUSTAL 2.1 multiple sequence alignment


WP_158320697.1_[B.subtilis]      ---------MTDLNISLLADMKLKELYELAKEYKISYYSKLTKKELIFSI
PPI97429.1_[E.coli]              ----------MNLSIAALENMKLKELYELAKEFKISYYSKLTKKELIFSI
AAW38423.1_[S.aureus]            MPERERTSPQYESFHELYKNYTTKELTQKAKTLKLTNYSKLNKKELVLAI
                                            :       : . *** : **  *:: ****.****:::*

WP_158320697.1_[B.subtilis]      LKARAEREGFFFMEGVLEIIQSEGFGFLRPINYTASSEDIYISASQIRRF
PPI97429.1_[E.coli]              LKARAEKEGFFFMEGVLEIIQSEGFGFLRPINYSPSSEDIYISASQIRRF
AAW38423.1_[S.aureus]            MEAQMEKDGNYYMEGILDDIQPDGYGFLRTVNYSKGEKDIYISASQIRRF
                                 ::*: *::* ::***:*: **.:*:****.:**: ..:************

WP_158320697.1_[B.subtilis]      DLRNGDKVSGKVRPPKENERYFGLLHVEAVNGADPESSKERVHFPALTPL
PPI97429.1_[E.coli]              DLRNGDKVSGKVRPPKENERYFGLLQVEAVNGDDPDSAKERVHFPALTPL
AAW38423.1_[S.aureus]            EIKRGDKVTGKVRKPKDNEKYYGLLQVDFVNDHNAEEVKKRPHFQALTPL
                                 :::.****:**** **:**:*:***:*: **. :.:. *:* ** *****

WP_158320697.1_[B.subtilis]      YPDRQIKLETTPTNLSTRIMDLVSPVGFGQRGLIVAPPKAGKTMLLKSIA
PPI97429.1_[E.coli]              YPDRQMKLETEPKKLPTRIMDLIAPVGFGQRGLIVAPPKAGKTSLLKEIA
AAW38423.1_[S.aureus]            YPDERIKLETETQNYSTRIMDLVTPIGLGQRGLIVAPPKAGKTSLLKEIA
                                 ***.::**** . : .******::*:*:*************** ***.**

WP_158320697.1_[B.subtilis]      NSITTNHPEAELIVLLIDERPEEVTDIERSVDA-DVVSSTFDEVPENHIK
PPI97429.1_[E.coli]              HSVTTNHPEAELIVLLIDERPEEVTDIERSVKG-DVVSSTFDEVPENHIK
AAW38423.1_[S.aureus]            NAISTNKPDAKLFILLVGERPEEVTDLERSVEAAEVVHSTFDEPPEHHVK
                                 ::::**:*:*:*::**:.********:****.. :** ***** **:*:*

WP_158320697.1_[B.subtilis]      VAELVLERAMRLVEHRRDVVILMDSITRLARAYNLVIPPSGRTLSGGIDP
PPI97429.1_[E.coli]              VAELVLERAMRLVEHKKDVIILMDSITRLARAYNLVIPPSGRTLSGGIDP
AAW38423.1_[S.aureus]            VAELLLERAKRLVEIGEDVIILMDSITRLARAYNLVIPPSGRTLSGGLDP
                                 ****:**** ****  .**:***************************:**

WP_158320697.1_[B.subtilis]      AAFHRPKRFFGAARNIEEGGSLTILATALVDTGSRMDDVIYEEFKGTGNL
PPI97429.1_[E.coli]              AAFHRPKRFFGAARNIEEGGSLTILATALVDTGSRMDDVIYEEFKGTGNM
AAW38423.1_[S.aureus]            ASLHKPKAFFGAARNIEAGGSLTILATALVDTGSRMDDMIYEEFKGTGNM
                                 *::*:** ********* ********************:**********:

WP_158320697.1_[B.subtilis]      ELHLDRSLAERRIFPAIDLQRSGTRKEELLIPKEQLDKLWKLRKAMSDTP
PPI97429.1_[E.coli]              ELHLDRSLAERRIFPAIDIRRSGTRKEDLLIPKEHLDKLWGIRKTMRDTP
AAW38423.1_[S.aureus]            ELHLDRKLSERRIFPAIDIGRSSTRKEELLISKSELDTLWQLRNLFTDST
                                 ******.*:*********: **.****:***.*..**.** :*: : *:.

WP_158320697.1_[B.subtilis]      DFMEKFMRKLKKSKTNEEFFNMLTEELKGRTNSKTLV-
PPI97429.1_[E.coli]              DFVESFLRKLRQTKTNEEFLQNIVADSKRYVTTK----
AAW38423.1_[S.aureus]            DFTERFIRKLKRSKNNEDFFKQLQKSAEESTKTGRPII
                                 ** * *:***:::*.**:*:: :  . :  ..:
```

Figure S2. Protein sequence alignment of Rho transcription termination factor. Alignment of the protein sequences of Rho transcription termination factor from *S. aureus*, *B. subtilis* and *E. coli.* Alignments were performed using CLUSTAL 2.1 multiple sequence alignment. The red box indicates the glycine residue substituted in the F9 mutant. The blue box indicates the phenylalanine residue directly involved in the interaction with RNA.

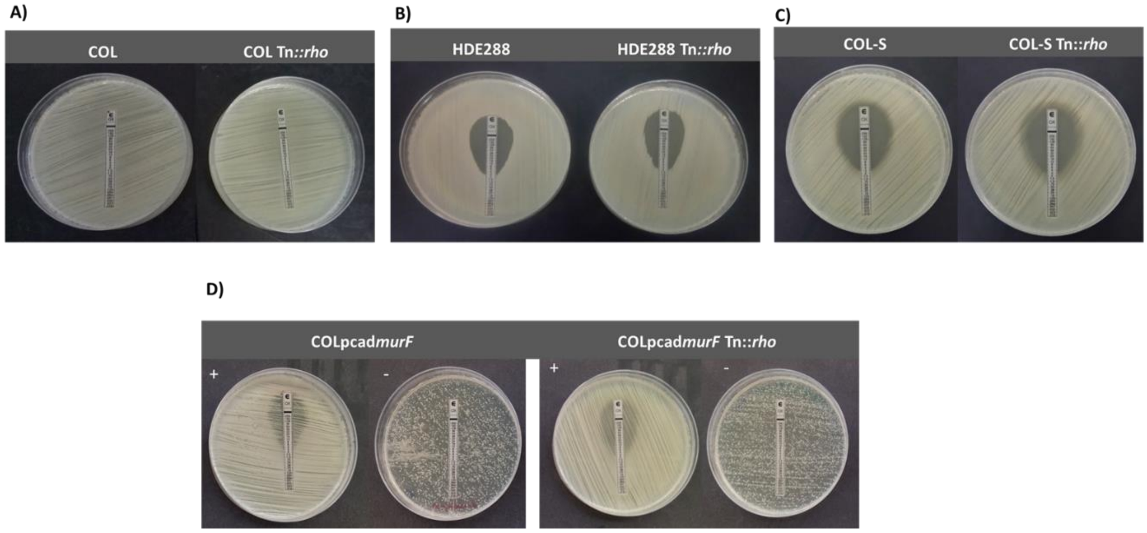

Figure S3. Analysis of oxacillin resistance of *rho* transposition mutants by E-test. A) strain COL (left panel) and its isogenic Tn::*rho* mutant (right panel); **B)** strain HDE288 (left panel) and its isogenic Tn::*rho* mutant (right panel); **C)** strain COL-S (left panel) and its isogenic Tn::*rho* mutant (right panel); **D)** strain COLpcad*murF* (left) and its isogenic double mutant COLpcad*murF* Tn::*rho* (right) in the presence (+) and in the absence (-) of inducer (0.2 µM $CdCl_2$).

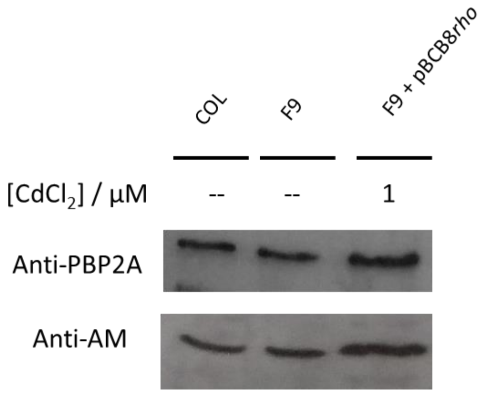

Figure S4. Western blotting of the membrane fraction of strain COL and mutants F9 and F9+pBCB8*rho* in the presence of 1µM of $CdCl_2$ using a polyclonal antibody raised against *S. aureus* PBP2A and a polyclonal antibody raised against the amidase (AM) domain of *S. aureus* Atl protein.

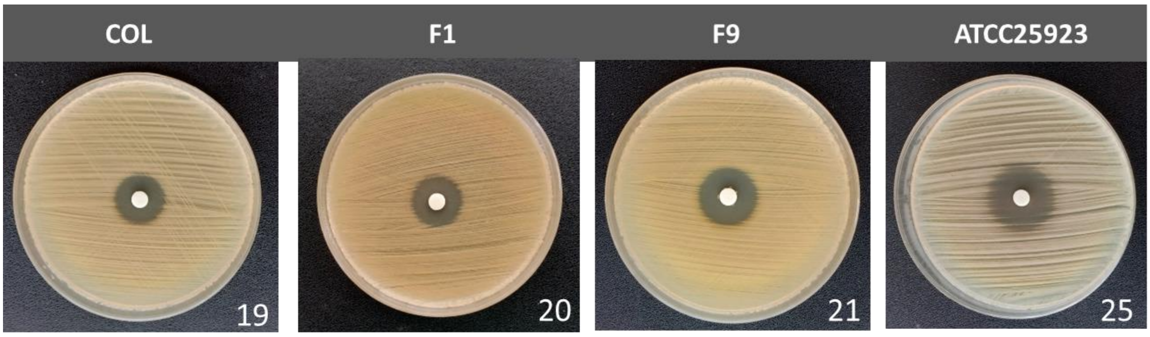

Figure S5. Analysis of ceftaroline resistance of insertion mutants. Growth inhibition halos of ceftaroline (30 µg) obtained by disk diffusion for the parental strain COL, the insertion mutants F1 and F9 and the ceftaroline susceptible control strain ATCC25923. The values presented correspond to the halo diameter in mm.

Table S1: List of strains and plasmids used in this study

Strain/Plasmid	Description	Source or Reference
E. coli		
DH5α	*recA endA1 gyrA*96 *thi*-1 *hsdR*17 *supE*44 *relA*1 Φ80 Δ*lacZ*ΔM15	Invitrogen
S. aureus		
COL	Homogeneous Mc^{r}, Em^{s}	Rockefeller University
RN4220	Mc^{s} , restriction negative	R. Novick
F1-F30	COL (*murF*::pRS2), Em^{r}	This study
F9	COL (*murF*::pRS2), Em^{r}	(1)
COLpcad*murF*	COL with *murF* gene under pcad control, Kan^{r}, Neo^{r}	(2)
COL-S	COL with SCCmec excised; Mc^{s}	(3)
HDE288	MRSA Low level Mc^{r} (Scc*mec*IV)	(4)
NE149	USA300-JE2 with Tn *bursa aurealis* insertion in *rho* gene	(5)
**Rho mutants:**		
RN4220 + pBCB8	RN4220 with replicative vector pBCB8	This study
RN4220 + pBCB8*rho*	RN4220 with *rho* under pcad control in replicative vector pBCB8, Kan^{r} , Neo^{r}	This study
COL+ pBCB8*rho*	COL with *rho* under pcad control in replicative vector pBCB8, Kan^{r} , Neo^{r}	This study
F9+ pBCB8*rho*	F9 (*murF*::pRS2) transductant with *rho* under pcad control in replicative vector pBCB8, Em^{r} , Kan^{r} , Neo^{r}	This study
F20 + pBCB8*rho*	F20 (*murF*::pRS2) transductant with *rho* under pcad control in replicative vector pBCB8, Em^{r} , Kan^{r} , Neo^{r}	This study
F26 + pBCB8*rho*	F26 (*murF*::pRS2) transductant with *rho* under pcad control in replicative vector pBCB8, Em^{r} , Kan^{r} , Neo^{r}	This study
COL + pBCB8	COL with replicative vector pBCB8 Kan^{r} , Neo^{r}	This study
F9 + pBCB8	F9 with replicative vector pBCB8 Em^{r}, Kan^{r} , Neo^{r}	This study
F20 + pBCB8	F20 with replicative vector pBCB8 Em^{r}, Kan^{r} , Neo^{r}	This study
F26 + pBCB8	F26 with replicative vector pBCB8 Em^{r}, Kan^{r} , Neo^{r}	This study
COL Tn::*rho*	COL with Tn *bursa aurealis* insertion in *rho* gene	This study
COLpcad*murF* Tn::*rho*	COLpcad*murF* with Tn *bursa aurealis* inserted on *rho* gene	This study
COL-S Tn::*rho*	COL-S with Tn *bursa aurealis* inserted inn *rho* gene	This study
HDE288 Tn::*rho*	HDE288 with Tn *bursa aurealis* inserted in *rho* gene	This study
**Plasmids**		
pBCB8	*S. aureus* replicative vector with pcad inducible promotor, Amp^{r}, Kan^{r}	R. Sobral and M. Pinho (unpublished)
pBCB20	*S. aureus* integrative vector with pcad inducible promotor, Ap^{r} , Kan^{r}	(6)

pBCB8*rho*	pBCB8 vector with *rho* rbs and *rho* fused to pcad promotor, Amp^{r} , Kan^{r}	This study

Table S2. Primers used in this study

Primer ID	Sequence 5'→3'	Source or Reference
**<u>Insertion mutants and confirmation of *murF* restoration</u>**		
**pmurFup4**	GTATGCGTATGGAACAACA	(1)
**p112**	GAAGTGGTAAATGCTTTAATTTCATAG	This study
**pucM13**	CGCCAGGGTTTTCCCAGTCACGAC	(7)
**<u>General cloning</u>**		
**pcadF**	GCACTTATTCAAGTGTATTT	R. Novick
**<u>Construction of *rho* overexpression mutants</u>**		
**p93**	TAAGGATCCAAATGGGTGTAAACTAATGC	This study
**p94**	GCCGGAATTCTATTAAATTATAGGTCGA	This study
**<u>Tn::*rho* insertion mutants</u>**		
**Upstream**	CTCGATTCTATTAACAAGGG	(5)
**Buster**	GCTTTTTCTAAATGTTTTTTAAGTAAATCAAGTAC	(5)
**p50**	AAAAATAATGAAATGGGTGTAAAC	This study
**p54**	TATTTTCAGCTCCTTTGCCC	This study
**<u>Northern blotting probe</u>**		
**pmurFnorthF**	CGCGAGGGGATTGCTAAAGC	This study
**pmurFnorthR**	TGCTTTTTCGACATGTTGC	This study

REFERENCES

1. Sobral RG, Ludovice AM, Gardete S, Tabei K, De Lencastre H, Tomasz A. 2003. Normally functioning murF is essential for the optimal expression of methicillin resistance in Staphylococcus aureus. Microb Drug Resist 9:231-41.
2. Figueiredo TA, Ludovice AM, Sobral RG. 2014. Contribution of peptidoglycan amidation to beta-lactam and lysozyme resistance in different genetic lineages of Staphylococcus aureus. Microb Drug Resist 20:238-49.
3. Pereira SF, Henriques AO, Pinho MG, de Lencastre H, Tomasz A. 2007. Role of PBP1 in cell division of Staphylococcus aureus. J Bacteriol 189:3525-31.
4. Rolo J, Miragaia M, Turlej-Rogacka A, Empel J, Bouchami O, Faria NA, Tavares A, Hryniewicz W, Fluit AC, de Lencastre H. 2012. High genetic diversity among community-associated Staphylococcus aureus in Europe: results from a multicenter study. PLoS One 7:e34768.
5. Fey PD, Endres JL, Yajjala VK, Widhelm TJ, Boissy RJ, Bose JL, Bayles KW. 2013. A genetic resource for rapid and comprehensive phenotype screening of nonessential Staphylococcus aureus genes. MBio 4:e00537-12.
6. Figueiredo TA, Sobral RG, Ludovice AM, Almeida JM, Bui NK, Vollmer W, de Lencastre H, Tomasz A. 2012. Identification of genetic determinants and enzymes involved with the amidation of glutamic acid residues in the peptidoglycan of Staphylococcus aureus. PLoS Pathog 8:e1002508.
7. Messing J. 1983. New M13 vectors for cloning. Methods Enzymol 101:20-78.
